# Prognostic Value of Cystatin C Across Ejection Fraction Spectrum in Heart Failure With Normal to Mild Renal Dysfunction Original Investigation

**DOI:** 10.1002/clc.70310

**Published:** 2026-04-20

**Authors:** Lyu Lyu, Juan Xu, Qiang Yang, Yun Wei, Benchuan Hao, Hongbin Liu

**Affiliations:** ^1^ Department of Cardiology, Beijing Anzhen Hospital Capital Medical University Beijing China; ^2^ Medical School of Chinese PLA Beijing China; ^3^ Department of General Surgery, Affiliated Xiaoshan Hospital Hangzhou Normal University Hangzhou China; ^4^ Department of Cardiology The Second Affiliated Hospital of Xi'an Jiaotong University Xi'an China; ^5^ Department of Cardiology The Ninth Medical Center, Chinese PLA General Hospital Beijing China; ^6^ Department of Cardiology The Second Medical Center, Chinese PLA General Hospital Beijing China

**Keywords:** cystatin C, heart failure with mid‐range ejection fraction, heart failure with preserved ejection fraction, heart failure with reduced ejection fraction, the Meta‐Analysis Global Group in Chronic Heart Failure Risk Score

## Abstract

**Background:**

Cystatin C (CysC)'s predictive utility for long‐term adverse outcomes in heart failure (HF) patients with normal to mild renal insufficiency remains unclear. This study investigates the relationship between CysC and adverse outcomes in HF patients across the whole ejection fraction (EF) spectrum with normal to mild renal insufficiency.

**Methods:**

In this single‐center cohort study, 637 HF patients with normal to mild renal insufficiency were categorized into reduced EF (HFrEF), mid‐range EF (HFmrEF), and preserved EF (HFpEF) groups. Associations between natural log unit (CysC) and risks of all‐cause mortality and HF rehospitalization were examined using Cox regression models. C‐index, IDI, and NRI assessed the incremental prognostic value.

**Results:**

Over a follow‐up of 9.4 years, 271 patients died, and 330 were rehospitalized for HF. Multivariate Cox regression analysis indicated significant associations between elevated CysC levels and increased risks of all‐cause mortality (HR = 1.99, 95% CI: 1.57–2.54) and HF rehospitalization (HR = 1.95, 95% CI: 1.57–2.42) across all patients. HR's for all‐cause mortality observed for HFmrEF (HR 4.73, 95% CI: 2.08−10.74) and HFrEF (HR 15.11, 95% CI: 6.24−36.60) were higher compared to those for HFpEF patients (HR = 1.48, 95% CI 0.98−2.23), and CysC showed less prognostic impact on all‐cause mortality in HFpEF patients. Including CysC in the MAGGIC risk score‐based model provided additional prognostic value for all subjects, even with N‐terminal pro‐brain natriuretic peptide (NT‐proBNP) levels added.

**Conclusions:**

CysC is an independent risk factor for adverse outcomes in HF patients across EF spectrum with normal to mild renal insufficiency. Integrating CysC into the MAGGIC risk score‐based model enhances its prognostic capability for predicting adverse outcomes in the general HF population. Its prognostic effect on all‐cause mortality is limited in HFpEF patients.

## Summary

- Our research is the first to demonstrate a significant correlation between Cystatin C levels and long‐term outcomes across all hospitalized heart failure patient types with normal to mildly impaired renal function, backed by an extensive follow‐up period. It is also the first to reveal the incremental prognostic benefit of adding Cystatin C to the MAGGIC risk score‐based model for all HF patient groups.
- Cystatin C proves to be a valuable biomarker for predicting the risk of adverse outcomes in heart failure patients with normal to mildly impaired renal function and coronary heart disease, based on a median follow‐up period of 9.4 years. However, in patients with heart failure with preserved ejection fraction, Cystatin C showed a reduced predictive value for all‐cause mortality.
- Incorporating Cystatin C into the MAGGIC risk score‐based model enhances its prognostic value for predicting adverse outcomes.

## Graphical abstract

Methods and cohort: HF patients with normal to mild renal insufficiency (*n* = 637), divided into HFpEF, HFmrEF, and HFrEF. Statistical Method: Cox regression models, Kaplan‐Meier Curves, C‐index, IDI, and NRI. Findings: Over a follow‐up of 9.4 years, significant associations of elevated CysC levels with increased risks of all‐cause mortality and HF rehospitalization across all patients were found. Including CysC in the MAGGIC risk score‐based model provided additional prognostic value for patients with normal to mild renal insufficiency. In subgroup analysis, comparable results were observed in HFmrEF and HFrEF patients. However, CysC showed less prognostic impact on all‐cause mortality in HFpEF patients (HR = 1.48, 95% CI: 0.98−2.23). Conclusions: CysC is an independent risk factor for adverse outcomes in HF patients across the EF spectrum with normal to moderate renal insufficiency. Integrating CysC into the MAGGIC risk score‐based model enhances its prognostic capability for predicting adverse outcomes in the general HF population, although its prognostic effect on all‐cause mortality is limited in HFpEF patients.

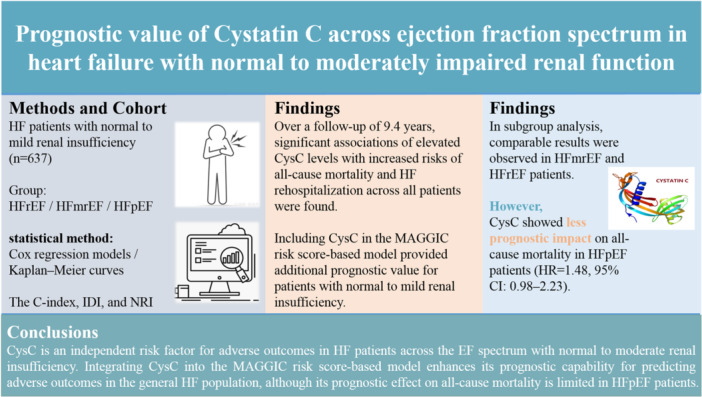

**Abbreviations**
ACE‐I: angiotensin‐converting enzyme inhibitors
AF: atrial fibrillation
ARB: angiotensin II receptor blockers
AVV max: maximum aortic valve velocity
BMI: body mass index
CABG: coronary artery bypass grafting
CCB: calcium channel blockers
CHD: coronary heart disease
CI: confidence interval
COPD: chronic obstructive pulmonary disease
CysC: Cystatin C
C‐index: concordance index
DBP: diastolic blood pressure
DM: diabetes mellitus
EF: ejection fraction
eGFR: estimated glomerular filtration rate
E/A: early (E) mitral inflow peak/atrial (A) filling peak ratio
HbA1C: hemoglobin A1c
HDL‐C: high‐density lipoprotein cholesterol
HF: heart failure
HFmrEF: heart failure with mid‐range ejection fraction
HFpEF: heart failure with preserved ejection fraction
HFrEF: heart failure with reduced ejection fraction
HR: hazard ratio
Hs‐TnT: high‐sensitivity cardiac troponin T
IDI: integrated discrimination improvement
IQR: interquartile range
IVC: inferior vena cava
IVST: interventricular septum thickness
LAD: left atrium dimension
LAVI: left atrial volume index
LDL‐C: low‐density lipoprotein cholesterol
LVEDD: left ventricular end‐diastolic dimension
LVEDV: left ventricular end‐diastolic volume
LVEF: left ventricular ejection fraction
LVESD: left ventricular end‐systolic diameter
LVESV: left ventricular end‐systolic volume
LVFS: left ventricular fraction shortening
LVMI: left ventricular mass index
LVPWT: left ventricular posterior wall thickness
MAGGIC risk score: the Meta‐Analysis Global Group in Chronic Heart Failure Risk Score
MDRD: Modification of Diet in Renal Disease
NRI: net reclassification improvement
NT‐proBNP: N‐terminal pro‐brain natriuretic peptide
NYHA‐FC: New York Heart Association Functional Class
PAP: pulmonary artery pressure
PCI: percutaneous coronary intervention
PY: person‐year
RAD: right atrial diameter
RCS: restricted cubic splines
RVD: right ventricular diameter
RVFW: right ventricular free wall
SBP: systolic blood pressure
SD: standard deviation
TC: total cholesterol
TRV max: maximum tricuspid regurgitation velocity

## 1 Introduction

Heart failure (HF) is becoming more prevalent as the population ages. As a significant cardiovascular disorder, HF poses a substantial challenge to human health and health care systems [1]. The classification of HF into distinct subtypes based on left ventricular ejection fraction (EF) is crucial, as the effectiveness of treatments and understanding of the underlying pathophysiology vary across EF categories [2, 3].

The prognostic value of various biomarkers in HF has been extensively studied, yet the significance of these factors in different HF subtypes remains uncertain. Renal insufficiency, widely recognized as a significant negative prognostic factor in HF patients, is highly prevalent [4, 5]. Cystatin C (CysC), a sensitive indicator of renal function, accurately reflects the glomerular filtration rate by being freely filtered through the glomerulus and reabsorbed in the renal tubules, without reentering the bloodstream [6, 7]. Increased levels of CysC have been linked to a higher risk of adverse outcomes in HF patients [8, 9, 10, 11, 12, 13, 14, 15, 16], particularly those with normal or mildly reduced renal function [8, 9, 11, 14, 16]. Nonetheless, the prognostic significance of CysC across all EF phenotypes in HF patients with normal or mild renal impairment has not been fully investigated. Our study seeks to evaluate the association between CysC levels and adverse outcomes across the full spectrum of EF in HF patients with normal to mild renal dysfunction and coronary heart disease (CHD).

Furthermore, the Meta‐Analysis Global Group in Chronic (MAGGIC) HF risk score [17] is considered a reliable method for predicting adverse outcomes in HF patients [18, 19, 20, 21]. This study aims to determine whether incorporating CysC into the MAGGIC risk score enhances the predictive accuracy of the model for adverse outcomes in our subjects.

## 2 Methods

### 2.1 Study Population

Between October 2010 and September 2014, we enrolled consecutive patients with HF and concurrent CHD from the Department of Cardiology at the Chinese PLA General Hospital in Beijing, China. All individuals have been hospitalized at least once or multiple times due to HF. The inclusion criteria were a diagnosis of HF based on the European Society of Cardiology guidelines [22]. The diagnostic criteria of CHD is at least one stenosis ≥ 50%, diagnosed by coronary arteriography. We classified patients into three categories: HF with reduced EF (HFrEF), HF with mid‐range EF (HFmrEF), and HF with preserved EF (HFpEF). We excluded patients who had moderate or severe valvular heart disease, severe pulmonary hypertension, arrhythmogenic right ventricular dysplasia, congenital heart disease, right ventricular infarction, pericardial disease, or specific cardiomyopathies; those with a life expectancy of less than 1 year; or those missing critical variables. After excluding 3 patients for missing key variables and 31 lost to follow‐up, 823 patients remained. We further excluded patients with an estimated glomerular filtration rate (eGFR) (calculated with the modified Modification of Diet in Renal Disease (MDRD) equation) of 60 mL/min per 1.73 m^2^ or less (*n* = 186), leaving 637 HF patients available for long‐term analysis, comprising 166 HFrEF, 236 HFmrEF, and 235 HFpEF patients. The study protocol was approved by the ethics committee of the Chinese PLA General Hospital, and all participants provided written informed consent at the initial visit.

## 3 Exposure Variable and Covariates

Serum CysC measured with immunoturbidimetric assay method at baseline was established as an exposure variable. Other laboratory indices were assessed using routine institutional laboratory procedures at the Chinese PLA General Hospital.

Clinical characteristics, discharge medications, and biochemical parameters were meticulously gathered from the patients' medical records. The assessments were performed by personnel who were unaware of the patients' initial characteristics and clinical outcomes.

CHD was recognized by the presence of ≥ 50% stenosis in at least one coronary artery through coronary arteriography. Hypertension was defined as having a systolic blood pressure (SBP) ≥ 140 mmHg, a diastolic blood pressure (DBP) ≥ 90 mmHg, or the use of antihypertensive medication. Diabetes mellitus (DM) was determined based on medical record diagnoses or by laboratory test results showing a hemoglobin A1c (HbA1c) level ≥ 6.5%. eGFR was calculated with MDRD equation: eGFR_MDRD_ $\left(\text{mL}/\min/1.73m^{2}\right)=175\times {\text{Scr}\;\left(\text{mg}/\text{dL}\right)}^{-1.234}\times {\text{Age}}^{-0.179}\times \;0.79\;\left(\text{if female}\right)$ [23]. eGFR_CysC_ was calculated with CKD‐EPI CysC equation (eGFR_CysC_ = 133 × (CysC/0.8)^−0.499^ × 0.996^Age^ [× 0.932 if female] [When CysC is less than 0.8]; eGFR_CysC_ = 133 × [CysC/0.8]^−1.328^ × 0.996^Age^ [×0.932 if female] [When CysC is greater than 0.8]) [24]. The MAGGIC risk score, derived from 13 variables, includes age, gender, body mass index (BMI), SBP, left ventricular EF (LVEF), creatinine level, current smoking status, DM, COPD, New York Heart Association classification, HF duration longer than 18 months, and the use of beta‐blockers and angiotensin‐converting enzyme inhibitors (ACEI) or angiotensin II receptor blockers (ARB) [17].

## 4 Outcomes and Follow‐up

The primary outcomes included all‐cause mortality and HF rehospitalization, monitored via biennial telephone interviews. These interviews involved participants or their proxies to gather data on hospitalizations or deaths occurring within the period, with the final follow‐up cut‐off in March 2023. Patients lacking recorded events by this deadline were classified as right‐censored for the analysis.

## 5 Statistical Analysis

Continuous variables were depicted as means with standard deviation (SD) or medians with interquartile range (IQR), based on their distribution; whereas, categorical variables were expressed as counts and percentages. For descriptive analysis, we utilized the Kruskal–Wallis and the chi‐square (*χ* ^2^) tests.

In survival analysis, we applied univariate Kaplan–Meier curves and multivariate Cox regression models to assess the relationship between CysC and adverse outcomes. The Kaplan–Meier curves underwent analysis via the log‐rank test. The multivariate Cox model assessed CysC as both a continuous and a categorical variable and was adjusted for MAGGIC risk score (Model 1) and for other risk factors including heart rate, MAGGIC risk score, hypertension, previous myocardial infarction, prior percutaneous coronary intervention (PCI)/coronary artery bypass grafting (CABG), stroke, anemia, atrial fibrillation (AF), chronic obstructive pulmonary disease (COPD), and medications such as statins, diuretics, spironolactone, digoxin, and calcium channel blockers (CCB), in addition to eGFR_MDRD_, low‐density lipoprotein cholesterol (LDL‐C), triglycerides, N‐terminal pro‐brain natriuretic peptide (NT‐proBNP), and high‐sensitivity cardiac troponin T (hs‐TnT) (Model 2). Variables including CysC, heart rate, MAGGIC risk score, LDL‐C, triglycerides, NT‐proBNP, and hs‐TnT underwent natural logarithm transformation. Furthermore, to explore the nonlinear relationship between CysC levels (as a continuous variable) and the outcomes of all‐cause mortality and HF rehospitalization, we employed restricted cubic splines (RCS) based on threshold analysis. To ascertain the incremental predictive value of incorporating CysC (as a continuous variable) into the MAGGIC risk score‐based model for forecasting risks of adverse outcomes, we calculated and compared the concordance index (C‐index), integrated discrimination improvement (IDI), and net reclassification improvement (NRI).

All statistical analyses were performed using R version 4.1.2 (The R Project for Statistical Computing, Vienna, Austria). A two‐tailed *p* value less than 0.05 was deemed statistically significant.

## 6 Results

### 6.1 Baseline Characteristics

Table 1 presents the baseline characteristics of 637 patients, categorized by CysC levels. The median age was 66.0 years (range: 56.0–75.0), with males making up 73.0% of the cohort. Patients with higher CysC levels were generally older and exhibited increased SBP, heart rate, creatinine, fasting glucose, HbA1c, NT‐proBNP, and hs‐TnT. This subgroup also showed a higher usage rate of diuretics, spironolactone, and digoxin, along with more common occurrences of DM, stroke, previous myocardial infarction, and AF. Conversely, these individuals had lower DBP, a smaller proportion of current smokers, and less frequent statin use. Echocardiographic measures, including left atrium dimension (LAD), interventricular septum thickness (IVST), left atrial volume index (LAVI), right ventricular diameter (RVD), right atrial diameter (RAD), inferior vena cava (IVC) size, maximum tricuspid regurgitation velocity (TRV), and pulmonary artery pressure (PAP), showed significant differences across the groups.

**Table 1:** Baseline characteristics of study patients according to status of Cystatin C.

Baseline characteristics	Total (*n* = 637)	Low Cystatin C (*n* = 212)	Medium Cystatin C (*n* = 213)	High Cystatin C (*n* = 212)	*p* value
Age, median (IQR), years	66.0 (56.0–75.0)	58.0 (50.0–67.0)	67.0 (58.0–76.0)	72.0 (62.0–80.0)	< 0.001
Male, *n* (%)	465 (73.0%)	163 (76.9%)	152 (71.4%)	150 (70.8%)	0.293
Current smokers, *n* (%)	183 (28.8%)	80 (37.7%)	60 (28.3%)	43 (20.3%)	< 0.001
Body mass index, mean (SD), kg/m²	24.9 ± 3.5	25.3 ± 3.3	24.9 ± 3.2	24.6 ± 3.9	0.149
Systolic blood pressure, median (IQR), mm Hg	130.0 (117.0–143.0)	130.0 (114.0–143.0)	129.0 (118.0–141.0)	134.0 (120.0–149.0)	0.029
Diastolic blood pressure, median (IQR), mm Hg	73.0 (65.0–80.0)	75.0 (68.0–82.0)	70.0 (64.0–78.0)	73.0 (65.0–83.0)	0.001
Heart rate, median (IQR), bpm	73.0 (66.0–82.0)	72.0 (65.0–80.0)	72.0 (65.0–80.0)	76.0 (68.0–85.0)	0.001
*NYHA‐FC, n (%)*					< 0.001
I/II	431 (67.7%)	173 (81.6%)	147 (69.0%)	111 (52.4%)	
III	154 (24.2%)	30 (14.2%)	51 (23.9%)	73 (34.4%)	
IV	52 (8.2%)	9 (4.2%)	15 (7.0%)	28 (13.2%)	
MAGGIC risk score, median (IQR), point	19.0 (14.0–25.0)	15.0 (12.0–19.0)	19.0 (15.0–25.0)	23.0 (18.0–27.0)	< 0.001
*Medical history*					
Diabetes mellitus, *n* (%)	241 (37.8%)	67 (31.6%)	79 (37.1%)	95 (44.8%)	0.019
Hypertension, *n* (%)	411 (64.5%)	129 (60.8%)	132 (62.0%)	150 (70.8%)	0.065
Previous myocardial infarction, *n* (%)	209 (32.8%)	56 (26.4%)	79 (37.1%)	74 (34.9%)	0.047
Previous PCI/CABG, *n* (%)	254 (39.9%)	88 (41.5%)	84 (39.4%)	82 (38.7%)	0.827
Stroke, *n* (%)	80 (12.6%)	15 (7.1%)	29 (13.6%)	36 (17.0%)	0.007
Anemia, *n* (%)	15 (2.4%)	2 (0.9%)	4 (1.9%)	9 (4.2%)	0.069
Chronic obstructive pulmonary disease, *n* (%)	58 (9.1%)	14 (6.6%)	23 (10.8%)	21 (9.9%)	0.286
Atrial fibrillation, *n* (%)	81 (12.7%)	13 (6.1%)	30 (14.1%)	38 (17.9%)	0.001
*Echocardiography*					
Left ventricular ejection fraction, median (IQR), %	46.0 (40.0–55.0)	45.0 (42.0–53.0)	46.0 (38.0–56.0)	46.0 (40.0–56.0)	0.633
LAD, median (IQR), mm	39.0 (35.0–42.0)	37.0 (34.0–41.0)	38.0 (35.0–42.0)	40.0 (36.0–43.0)	0.003
LVPWT, median (IQR), mm	10.0 (10.0–11.0)	10.0 (10.0–11.0)	10.0 (10.0–11.0)	10.0 (10.0–11.0)	0.967
LVEDD, median (IQR), mm	48.0 (45.0–54.0)	49.0 (45.0–53.0)	48.0 (45.0–53.0)	48.0 (44.0–56.0)	0.903
IVST, median (IQR), mm	11.0 (10.0–12.0)	11.0 (10.0–11.0)	11.0 (10.0–12.0)	11.0 (10.0–12.0)	0.015
LVMI, median (IQR), g/m^2^	105.4 (90.9–125.7)	105.0 (90.9–119.2)	105.0 (90.7–126.9)	107.7 (92.1–131.8)	0.209
LAVI, median (IQR), mL/m^2^	45.0 (35.1–58.0)	41.4 (33.9–50.0)	45.1 (35.7–59.1)	49.1 (36.8–62.4)	< 0.001
LVESD, median (IQR), mm	36.0 (31.0–41.0)	36.0 (32.0–40.0)	35.0 (31.0–40.0)	35.0 (32.0–43.0)	0.847
LVEDV, median (IQR), mm	110.0 (88.0–138.0)	112.0 (90.0–132.0)	110.0 (87.0–135.0)	108.0 (88.0–147.0)	0.907
LVESV, median (IQR), mm	58.0 (41.0–81.0)	58.0 (44.0–74.0)	57.0 (41.0–82.0)	58.0 (39.0–90.0)	0.986
LVFS, median (IQR), mm	26.0 (21.0–30.0)	26.0 (22.0–30.0)	26.0 (20.0–30.0)	26.0 (20.0–30.0)	0.325
RVD, median (IQR), mm	35.0 (32.0–37.0)	34.0 (31.0–37.0)	35.0 (32.0–38.0)	35.0 (32.0–38.0)	0.012
RAD, median (IQR), mm	34.0 (31.0–37.0)	34.0 (31.0–37.0)	35.0 (32.0–37.0)	35.0 (32.0–39.0)	0.045
RVFW, median (IQR), mm	5.0 (5.0–6.0)	5.0 (5.0–6.0)	6.0 (5.0–6.0)	6.0 (5.0–6.0)	0.741
IVC, median (IQR), mm	15.0 (14.0–17.0)	15.0 (14.0–16.0)	15.0 (14.0–16.0)	15.0 (14.0–17.0)	0.020
E/A, median (IQR)	0.8 (0.6–1.3)	0.8 (0.7–1.2)	0.8 (0.6–1.2)	0.8 (0.6–1.6)	0.372
TRV max, median (IQR), m/s	2.3 (2.1–2.6)	2.3 (2.1–2.5)	2.3 (2.1–2.6)	2.5 (2.1–2.9)	< 0.001
AVV max, median (IQR), m/s	1.2 (1.0–1.4)	1.2 (1.0–1.3)	1.2 (1.0–1.4)	1.2 (1.0–1.5)	0.718
PAP, median (IQR), mm Hg	29.0 (22.0–36.0)	26.0 (20.0–32.0)	29.0 (24.0–35.0)	32.0 (24.0–40.0)	< 0.001
*Medication*					
Statin, *n* (%)	592 (92.9%)	199 (93.9%)	205 (96.2%)	188 (88.7%)	0.008
Beta blocker, *n* (%)	496 (77.9%)	172 (81.1%)	161 (75.6%)	163 (76.9%)	0.355
ACE‐I/ARB, *n* (%)	330 (51.8%)	109 (51.4%)	103 (48.4%)	118 (55.7%)	0.318
Diuretic, *n* (%)	202 (31.7%)	42 (19.8%)	66 (31.0%)	94 (44.3%)	< 0.001
Spironolactone, *n* (%)	244 (38.3%)	57 (26.9%)	87 (40.8%)	100 (47.2%)	< 0.001
Digoxin, *n* (%)	86 (13.5%)	15 (7.1%)	26 (12.2%)	45 (21.2%)	< 0.001
Calcium channel blocker, *n* (%)	151 (23.7%)	50 (23.6%)	45 (21.1%)	62 (29.2%)	0.067
*Laboratory indicators*					
Creatinine, median (IQR), mg/dL	0.9 (0.8–1.0)	0.8 (0.7–0.9)	0.9 (0.8–1.0)	0.9 (0.8–1.1)	< 0.001
Glucose, median (IQR), mmol/L	6.4 (5.2–8.5)	6.0 (5.1–7.9)	6.7 (5.1–8.4)	6.5 (5.3–9.8)	0.039
HbA1c, median (IQR), %	6.2 (5.7–7.2)	6.1 (5.7–6.9)	6.2 (5.7–7.2)	6.4 (5.8–7.5)	0.005
HDL‐C, median (IQR), mmol/L	1.0 (0.8–1.2)	1.0 (0.8–1.2)	1.0 (0.9–1.2)	1.0 (0.9–1.2)	0.942
LDL‐C, median (IQR), mmol/L	2.2 (1.7–2.9)	2.2 (1.8–2.8)	2.2 (1.7–2.9)	2.3 (1.8–3.0)	0.674
Total cholesterol, median (IQR), mmol/L	3.8 (3.2–4.5)	3.8 (3.3–4.5)	3.7 (3.2–4.5)	3.9 (3.3–4.6)	0.438
Triglycerides, median (IQR), mmol/L	1.3 (0.9–1.8)	1.3 (0.9–1.8)	1.3 (0.9–1.9)	1.3 (0.9–1.8)	0.772
NT‐proBNP, median (IQR), pg/mL	822.0 (290.0–2,069.0)	530.5 (205.8–1,212.0)	775.0 (320.0–2,041.0)	1,265.0 (498.8–3,414.5)	< 0.001
hs‐TnT, median (IQR), ng/L	0.02 (0.01‐0.12)	0.02 (0.01‐0.18)	0.02 (0.01‐0.09)	0.03 (0.01‐0.12)	< 0.001
eGFR_MDRD_, mean (SD), mL/min/1.73 m^2^	99.4 ± 76.2	109.2 ± 24.1	91.9 ± 21.8	97.2 ± 127.6	0.056
eGFR_CysC_, mean (IQR), mL/min/1.73 m^2^	58.92 (43.79–80.37)	87.21 (78.65–95.36)	55.32 (49.14–63.33)	37.46 (30.74–41.87)	< 0.001
Cystatin C, median (IQR), mg/L	1.3 (1.2–1.6)	0.9 (0.8–1.0)	1.3 (1.1–1.3)	1.7 (1.5–1.9)	< 0.001

Abbreviations: ACE‐I, Angiotensin‐converting enzyme inhibitor; ARB, Angiotensin II receptor blocker; AVV, Aortic valve velocity; CABG, coronary artery bypass grafting; E/A, early (E) mitral inflow peak/atrial (A) filling peak ratio; eGFR, estimated glomerular filtration rate; HbA1c, Hemoglobin A1c; HDL‐C, High‐density lipoprotein cholesterol; hs‐TnT, high‐sensitivity cardiac troponin T; IQR, Inter‐quartile range; IVC, Inferior vena cava; IVST, Interventricular septum thickness; LAD, left atrium dimension; LAVI, Left atrial volume index; LDL‐C, low‐density lipoprotein cholesterol; LVEDD, Left ventricular end‐diastolic dimension; LVEDV, Left ventricular end‐diastolic volume; LVESD, Left ventricular end systolic diameter; LVESV, Left ventricular end‐systolic volume; LVFS, Left ventricular fraction shortening; LVMI, Left ventricular mass index; LVPWT, Left ventricular posterior wall thickness; MAGGIC risk score, The Meta‐Analysis Global Group in Chronic Heart Failure risk score; MDRD, Modification of Diet in Renal Disease; NT‐proBNP, N‐terminal pro‐brain natriuretic peptide; NYHA‐FC, New York Heart Association functional class; PAP, Pulmonary artery pressure; PCI, Percutaneous coronary intervention; RAD, right atrial diameter; RVD, right ventricular diameter; RVFW, Right ventricular free wall; TRV, tricuspid regurgitation velocity.

## 7 Cystatin C and All‐Cause Mortality

**Figure 1:**
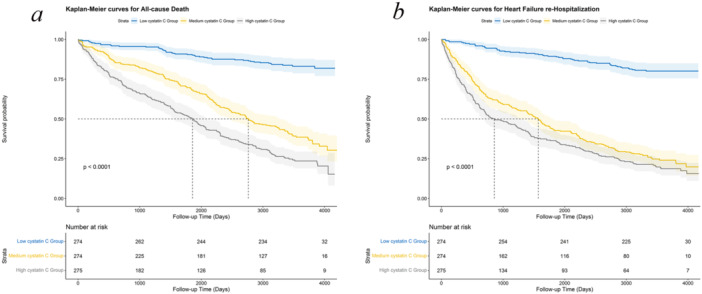
Kaplan–Meier survival curves for all‐cause mortality and HF rehospitalizition in all HF patients. Kaplan–Meier survival curves for (a) all‐cause mortality and (b) HF rehospitalizitions. HF, heart failure.

During a median follow‐up of 9.4 years (range: 8.7–11.5), 271 patients died. Kaplan–Meier survival analysis curves depicted in Figure 1a illustrated the cumulative survival probabilities for all‐cause mortality across three groups categorized by CysC levels. The group with higher CysC levels showed a significantly increased rate of all‐cause mortality compared with patients exhibiting lower CysC levels (*p* < 0.001). The RCS curve within the Cox proportional hazards model revealed an increase in mortality risk associated with elevated CysC levels (a continuous variable), demonstrating a nonlinear relationship (*p* for nonlinearity = 0.003; Supporting Information S1: Figure 1a). Univariate Cox model analysis revealed a significant association between CysC levels and all‐cause mortality (per ln unit increase, hazard ratio [HR] = 2.51, 95% confidence interval [CI]: 2.13–2.96). This relationship was sustained in the multivariate model (per ln unit increase, model 1: HR = 1.97, 95% CI: 1.58–2.46; model 2: HR = 1.99, 95% CI: 1.57–2.54). Further division of CysC levels into high, medium, and low categories indicated that both high (model 1: HR = 4.57, 95% CI: 3.02–6.91; model 2: HR = 4.23, 95% CI: 2.79–6.42) and medium (model 1: HR = 2.46, 95% CI: 1.61–3.75; model 2: HR = 2.38, 95% CI: 1.56–3.64) CysC groups faced significantly higher risks of all‐cause mortality compared to the low CysC group (Table 2).

**Table 2:** Association between Cystatin C and adverse outcomes in heart failure patients.

Variables	Incidence per 1000 PYs (95% CI)	Unadjusted HR (95% CI)	Model 1^a^ Adjusted HR (95% CI)	Model 2^b^ adjusted HR (95% CI)	*p* for trend
All‐cause mortality	213.76 (189.77–240.79)				
Cystatin C (per natural logarithm unit)		2.51 (2.13–2.96)	1.97 (1.58–2.46)	1.99 (1.57–2.54)	
Low Cystatin C group	239.54 (167.48–342.60)	Ref.	Ref.		< 0.001
Medium Cystatin C group	171.57 (140.32–209.78)	3.59 (2.38–5.41)	2.46 (1.61–3.75)	2.38 (1.56–3.64)	
High Cystatin C group	247.95 (210.82–291.62)	7.83 (5.28–11.62)	4.57 (3.02–6.91)	4.23 (2.79–6.42)	
Heart failure rehospitalization	275.90 (247.69–307.34)				
Cystatin C (per natural logarithm unit)		2.41 (2.08–2.79)	1.92 (1.58–2.35)	1.95 (1.57–2.42)	
Low Cystatin C group	218.14 (160.01–297.40)	Ref.	Ref.		< 0.001
Medium Cystatin C group	268.84 (225.61–320.35)	4.25 (2.97–6.07)	3.40 (2.36–4.89)	3.35 (2.32–4.83)	
High Cystatin C group	301.24 (258.61–350.89)	7.19 (5.08–10.17)	4.61 (3.18–6.68)	4.58 (3.15–6.65)	

Abbreviations: CABG, coronary artery bypass grafting; CI, Confidence interval; eGFR, estimated glomerular filtration rate; HR, Hazard ratio; hs‐TnT, high‐sensitivity cardiac troponin T; LDL‐C, low‐density lipoprotein cholesterol; MAGGIC risk score, The Meta‐Analysis Global Group in Chronic Heart Failure risk score; MDRD, Modification of Diet in Renal Disease; NT‐proBNP, N‐terminal pro‐brain natriuretic peptide; PCI, Percutaneous coronary intervention; PY, Person‐year.

^a^ Model 1 adjusted for MAGGIC risk score.

^b^ Model 2 adjusted for heart rate; MAGGIC risk score; hypertension; previous myocardial infarction; previous PCI/CABG; stroke; anemia; atrial fibrillation; chronic obstructive pulmonary disease; statin; diuretic; spironolactone; digoxin; calcium channel blocker; eGFR_MDRD_; LDL‐C; triglycerides; NT‐proBNP, hs‐TnT.

## 8 CysC and HF Rehospitalization

During the follow‐up period, 330 patients experienced HF rehospitalization. The incidence of HF rehospitalization increased progressively with higher levels of CysC (*p* < 0.001, Figure 1b). The association between CysC levels (treated as a continuous variable) and HF rehospitalization was analyzed using a RCS approach, revealing a nonlinear relationship between elevated CysC levels and the risk of HF rehospitalization (*p* for nonlinearity < 0.001; Supporting Information S1: Figure 1b). Univariate Cox regression analysis showed a significant association between increased CysC levels (per natural log unit, HR = 2.41, 95% CI: 2.08–2.79) and a higher risk of HF rehospitalization. Multivariate analysis, after adjusting for confounders, confirmed CysC as a significant predictor of HF rehospitalization risk (per natural log unit, model 1: HR = 1.92, 95% CI: 1.58–2.35; model 2: HR = 1.95, 95% CI: 1.57–2.42). Additionally, categorization of CysC levels showed that patients with high (model 1: HR = 4.61, 95% CI: 3.18–6.68; model 2: HR = 4.58, 95% CI: 3.15–6.65) and medium (model 1: HR = 3.40, 95% CI: 2.36–4.89; model 2: HR = 3.35, 95% CI: 2.32–4.83) CysC levels had significantly increased risks of HF rehospitalization compared to those in the low CysC group (Table 2).

## 9 Incremental Value of CysC in Predicting Adverse Outcomes

C‐statistics, NRI, and IDI were used to further evaluate whether the MAGGIC risk score‐based model was improved by incorporating CysC.

Table 3 shows that adding CysC to the MAGGIC risk score‐based model significantly enhanced discrimination for both all‐cause mortality (Δ C‐index = 0.025, 95% CI: 0.005–0.045; IDI = 0.031, 95% CI: 0.002–0.056; continuous NRI = 0.300, 95% CI: 0.051–0.474) and HF rehospitalization (Δ C‐index = 0.031, 95% CI: 0.003–0.059; IDI = 0.044, 95% CI: 0.007–0.089; continuous NRI = 0.456, 95% CI: 0.242–0.622) across all HF patients.

**Table 3:** Incremental value of Cystatin C for predicting adverse outcomes in all heart failure patients.

Models	C‐index (95% CI)	∆ C‐index^a^ (95% CI)	IDI^a^ (95% CI)	continuous NRI^a^ (95% CI)
*All‐cause mortality*				
MAGGIC risk score	0.718 (0.687–0.749)	—	—	—
MAGGIC risk score + Cystatin C	0.743 (0.714–0.772)	0.025 (0.005–0.045)	0.031 (0.002–0.056)	0.300 (0.051–0.474)
*Heart failure rehospitalization*				
MAGGIC risk score	0.701 (0.674–0.728)	—	—	—
MAGGIC risk score + Cystatin C	0.732 (0.705–0.759)	0.031 (0.003–0.059)	0.044 (0.007–0.089)	0.456 (0.242–0.622)

Abbreviations: CI, Confidence interval; IDI, Integrated discrimination improvement; MAGGIC risk score, The Meta‐Analysis Global Group in Chronic Heart Failure risk score; NRI, Net reclassification index.

^a^ MAGGIC risk score versus MAGGIC risk score + Cystatin C.

## 10 CysC and Subgroup Analyses of Adverse Outcomes

**Figure 2:**
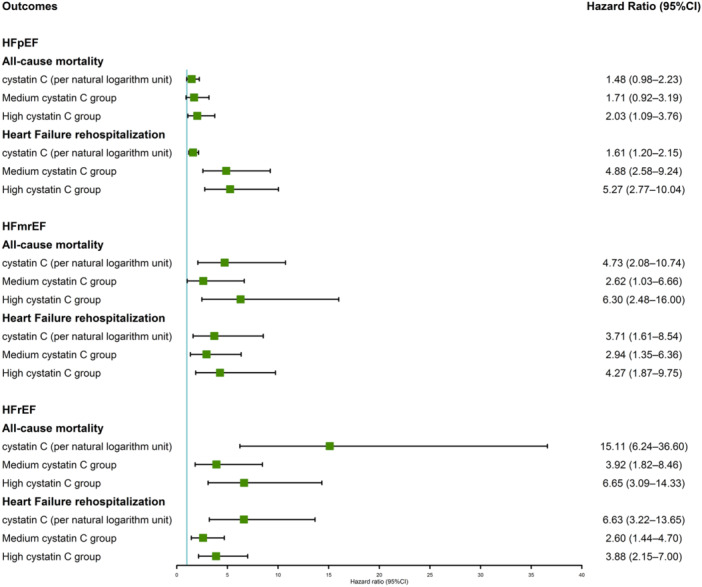
Association between Cystatin C and adverse outcomes in different subgroups of heart failure. Multivariable model adjusted for heart rate; MAGGIC risk score; hypertension; previous myocardial infarction; previous PCI/CABG; stroke; anemia; atrial fibrillation; chronic obstructive pulmonary disease; statin; diuretic; spironolactone; digoxin; calcium channel blocker; eGFR_MDRD_; LDL‐C; triglycerides; NT‐proBNP, hs‐TnT. CABG, coronary artery bypass grafting; CI, confidence interval; eGFR, estimated glomerular filtration rate; HR, hazard ratio; hs‐TnT, high‐sensitivity cardiac troponin T; LDL‐C, low‐density lipoprotein cholesterol; MAGGIC risk score, the Meta‐Analysis Global Group in Chronic Heart Failure risk score; MDRD, modification of diet in renal disease; NT‐proBNP, N‐terminal pro‐brain natriuretic peptide; PCI, percutaneous coronary intervention.

We investigated the associations between CysC levels and adverse outcomes across different HF subtypes. Our analyses of HFrEF and HFmrEF showed consistent associations with all‐cause mortality. Specifically, higher CysC levels were linked to an increased risk of all‐cause mortality in both HFmrEF and HFrEF groups (adjusted model: HFmrEF: per ln unit increase, HR = 4.73, 95% CI: 2.08–10.74; medium CysC group, HR = 2.62, 95% CI: 1.03–6.66; high CysC group, HR = 6.30, 95% CI: 2.48–16.00; HFrEF: per ln unit increase, HR = 15.11, 95% CI: 6.24–36.60; medium CysC group, HR = 3.92, 95% CI: 1.82–8.46; high CysC group, HR = 6.65, 95% CI: 3.09–14.33; Figure 2). However, upon categorizing CysC levels into high, medium, and low within HFpEF patients, a significant association with all‐cause mortality was only noted in the high CysC group (HR = 2.03, 95% CI: 1.09–3.76), with no significant association observed for CysC as a continuous variable (HR = 1.48, 95% CI: 0.98–2.23; Figure 2). *p* value of interaction between age and CysC for all‐cause death in HFpEF, HFmrEF, and HFrEF were 0.43, 0.96, and 0.002 respectively, and *p* value of interaction of age with CysC for HF readmission in HFpEF, HFmrEF, and HFrEF were 0.28, 0.40, and 0.001 respectively. Owing to significant interaction of age with CysC for adverse outcomes in HFrEF patients, we divided HFrEF patients into two groups based on age and analyzed the prognostic role of CysC in both groups. The results showed that patients over 65 years old was associated with a lower HR value than those under 65 years old (Supporting Information S1: Figure 2).

Subgroup analyses focusing on HF rehospitalization across different HF categories yielded similar results. Significant associations between CysC levels and the risk of HF rehospitalization were observed across all HF categories (adjusted model: HFpEF: per ln unit increase, HR = 1.61, 95% CI: 1.20–2.15; medium CysC group, HR = 4.88, 95% CI: 2.58–9.24; high CysC group, HR = 5.27, 95% CI: 2.77–10.04; HFmrEF: per ln unit increase, HR = 3.71, 95% CI: 1.61–8.54; medium CysC group, HR = 2.94, 95% CI: 1.35–6.36; high CysC group, HR = 4.27, 95% CI: 1.87–9.75; HFrEF: per ln unit increase, HR = 6.63, 95% CI: 3.22–13.65; medium CysC group, HR = 2.60, 95% CI: 1.44–4.70; high CysC group, HR = 3.88, 95% CI: 2.15–7.00; Figure 2).

## 11 Associations between Different Renal Function Markers and Adverse Outcomes

We demonstrated the prognostic role of different renal function markers in overall population and in subgroups HF patients. In total cohort individuals, multivariate Cox regression represented that only CysC (per natural log unit, model 2: All‐cause mortality: HR = 5.10, 95% CI: 2.86–9.07; HF rehospitailization: HR = 5.04, 95% CI: 3.03–8.38) and eGFR_CysC_ (per natural log unit, model 2: All‐cause mortality: HR = 0.28, 95% CI: 0.18–0.43; HF rehospitailization: HR = 0.27, 95% CI: 0.18–0.40) can predict both the risk of all‐cause mortality and HF readmission in the overall population. eGFR_MDRD_ (per natural log unit, model 2: All‐cause mortality: HR = 0.67, 95% CI: 0.34–1.31; HF rehospitailization: HR = 0.39, 95% CI: 0.21–0.72) and creatinine (per natural log unit, model 2: All‐cause mortality: HR = 1.92, 95% CI: 0.89–4.12; HF rehospitailization: HR = 2.67, 95% CI: 1.34–5.29) can significantly predict HF rehospitailization, but not all‐cause death (Supporting Information S1: Table 2).

Similar results were shown in HFmrEF and HFrEF patients, only CysC (per natural log unit, model 2: HFmrEF patients: All‐cause mortality: HR = 10.30, 95% CI: 3.60–29.48; HF rehospitailization: HR = 5.61, 95% CI: 2.49–12.61; HFrEF patients: All‐cause mortality: HR = 2.37, 95% CI: 1.03–2.51; HF rehospitailization: HR = 2.15, 95% CI: 1.09–4.30) and eGFR_CysC_ (per natural log unit, model 2: HFmrEF patients: All‐cause mortality: HR = 0.17, 95% CI: 0.07–0.37; HF rehospitailization: HR = 0.27, 95% CI: 0.15–0.50; HFrEF patients: All‐cause mortality: HR = 0.41, 95% CI: 0.18–0.97; HF rehospitailization: HR = 0.45, 95% CI: 0.21–0.97) represented prognostic value for both all‐cause mortality and HF readmission in patients with HFmrEF and HFrEF (Supporting Information S1: Table 3).

In HFpEF patients, the results of multivariate Cox regression showed that all renal function factors cannot predict the risk of all‐cause mortality Except for creatinine, significant associations were observed between other renal function markers and the risk of HF rehospitailization in HFpEF patients (per natural log unit, CysC: model 2: HR = 1.71, 95% CI: 1.30–2.26; CysC‐based eGFR: model 2: HR = 0.67, 95% CI: 0.54–0.83; eGFR_MDRD_: model 2: HR = 0.40, 95% CI: 0.17–0.96; creatinine: model 2: HR = 2.16, 95% CI: 0.78–5.99; Supporting Information S1: Table 2, 3).

Meanwhile, the Spearman correlation coefficient between logarithmic values of eGFR_MDRD_ and eGFR_CysC_ was 0.50 (*p* < 0.0001) in total cohort, 0.53 (*p* < 0.0001) in HFpEF patients, 0.44 (*p* < 0.0001) in HFmrEF patients, and 0.46 (*p* < 0.0001) in HFrEF patients.

## 12 Discussion

In this study, we observed a significant association between baseline CysC levels and adverse outcomes, including all‐cause mortality and HF rehospitalization, over an extended period among HF patients across all EF phenotypes with normal to mild renal dysfunction and CHD. Furthermore, incorporating CysC into assessments enhanced the discriminative capability beyond what the MAGGIC risk score‐based model provides for predicting long‐term adverse outcomes in our cohort. Subgroup analysis of different HF types revealed significance associations of CysC with adverse outcomes in patients with HFmrEF and HFrEF. However, CysC exhibited a nonsignificant prognostic impact on all‐cause mortality in patients with HFpEF, while it remained a significant predictor of HF rehospitalization in this group. The incremental effect of CysC into MAGGIC risk score‐based model for all‐cause mortality was no longer valid for all HF patients, but only for HFmrEF and HFrEF patients.

## 13 CysC and Adverse Outcomes in HF Patients

It is widely recognized that CysC serves as a sensitive and accurate biomarker for assessing renal insufficiency [6], identified as a significant adverse factor in HF [4, 5], signaling poorer hemodynamic status and a worsened prognosis. However, earlier research suggested that CysC is particularly beneficial for detecting early nephropathy in patients with a normal eGFR, highlighting its utility as an early marker of renal impairment [6]. Furthermore, Dupont et al. proposed that the prognostic significance of CysC primarily applies to individuals with relatively “preserved” renal function, while its utility appears limited in HF patients with already impaired renal function [9]. Thus, elevated CysC probably has a substantial effect on prognosis for patients with minor to moderate decline in renal function which is not detected by creatinine. Consequently, our study concentrates on the predictive role of CysC in HF patients with normal to mild renal insufficiency. Several cohort studies have demonstrated a significant link between CysC levels and an increased risk of adverse outcomes in HF patients with normal to mildly impaired renal function, encompassing acute HF [8, 16], stable chronic HF [9], and patients with HFpEF [14]. A meta‐analysis involving HF patients indicated that elevated CysC levels might be associated with an increased risk of all‐cause mortality and hospital readmission, independent of eGFR [11]. Nonetheless, these studies did not cover all EF phenotypes. Our research supports previous findings that CysC is a reliable and valuable biomarker for predicting all‐cause mortality and HF rehospitalization in our cohort, including the full spectrum of EF phenotypes in HF patients with normal to mildly impaired renal function and CHD over an extended follow‐up period. Compared to creatinine and eGFR_MDRD_, CysC is a better prognostic predictor in our cohort (Supporting Information S1: Table 2, 3), which meant CysC does something on its own, independently of being a renal function parameter. Therefore, the enhanced predictive value of CysC in HF patients with normal or mild renal insufficiency may be explained as follows: Firstly, elevated CysC levels may induce myocardial fibrosis by promoting collagen I production and altering collagen metabolism, leading to diastolic dysfunction [25, 26]. Secondly, changes in the balance between lysosomal cathepsins and endogenous inhibitors like CysC might facilitate cardiocyte remodeling and atherogenesis through inflammatory cytokines [27]. Thirdly, as an inhibitor of elastolytic cysteine proteases, CysC is implicated in the progression of atherosclerosis [28], contributing to further cardiac remodeling and dysfunction. Moreover, we observed the strong association of age with higher CysC concentrations among HF patients (Table 1), which was in line with previous studies [29, 30, 31]. The risk of localized or diffuse myocardial injury increases with age, leading to the occurrence or worsening of HF.

## 14 Comparison of Prognostic Role of CysC in Subtypes of HF

Subgroup analysis revealed similar associations between elevated CysC levels and a heightened risk of adverse outcomes in patients with HFmrEF and HFrEF. Age showed interaction with CysC in HFrEF patients, and we conducted a stratified subgroup analysis of the HFrEF patients by age (Supporting Information S1: Figure 2). The results from Supporting Information S1: Figure 2 represented that there was no significant difference in HR values among different age groups in HFrEF patients, mainly because of the large CIs of the HR. With older age, CysC does not discriminate as much as predictor of adverse outcomes. In patients with HFpEF, CysC proved to be a valuable prognostic biomarker for rehospitalization due to HF, while its prognostic significance for all‐cause mortality in HFpEF patients was less pronounced. As mentioned above in the possible reasons for predictive value of CysC in HF patients with normal or mild renal insufficiency, elevated CysC levels can lead to diastolic dysfunction [25, 26], which may be associated with left ventricle diameter or with left ventricular hypertrophy. Hence, the predictive value of CysC for all‐cause death in HFmrEF and HFrEF patients is better than that of HFpEF. In line with our study, two more studies confirm the diminished value fo CysC for the subgroup of HFpEF patients [32, 33], though not uniformly across all cohort studies [14, 15]. There are two other studies that still find some prognostic role for CysC in those with acute admissions for HFpEF and then primarily for the higher CysC quartile [14]. The referenced cohort defined HFpEF diagnosis with a LVEF ≥ 45%, inadvertently including some HFmrEF patients. Moreover, it did not assess CysC as a continuous variable in a multivariate Cox model; our research addressed this gap, demonstrating limited predictive value of CysC (as a continuous variable) in predicting all‐cause mortality in HFpEF patients. Another study identified CysC as a critical prognostic marker for all‐cause mortality in HFpEF as well as HFmrEF and HFrEF [15], encompassing ambulatory patients managed in a multidisciplinary HF clinic, contrasting our focus on hospitalized patients with more definitive diagnoses. Meanwhile, there is no significant interaction between age and CysC for all‐cause death in HFpEF patients. Age may explain more than CysC for all‐cause mortality in HFpEF patients in our cohort.

Our study suggests that CysC is a promising prognostic biomarker in HF patients. The incorporation of CysC into the MAGGIC‐based model enhances its incremental prognostic value in HF patients with normal to mild renal dysfunction and CHD. By combining CysC with clinical features and established risk factors, we enhance risk stratification in these patients.

## 15 Study Strengths and Limitations

This research is the first to demonstrate a significant correlation between CysC levels and long‐term outcomes across all hospitalized HF patient types with normal to mild renal dysfunction, backed by an extensive follow‐up period. It is also the first to reveal the incremental prognostic benefit of adding CysC to the MAGGIC risk score‐based model for all HF patient groups. Subgroup analyses indicated a strong association between CysC and adverse outcomes in patients with HFmrEF and HFrEF. Nonetheless, in patients with HFpEF, CysC showed nonsignificant predictive value for all‐cause mortality.

However, our study has limitations. Being a single‐center study of insured adults in China, our results may not extend to other demographics. Additionally, as our cohort includes HF patients with CHD, the findings may not be universally relevant to all HF populations. The study also encompasses a moderate sample size of HF patients with normal to mild renal dysfunction, potentially introducing selection bias. Moreover, our study chose HF patients with normal to mild renal dysfunction to verify the prognostic effect of CysC on HF besides renal insufficiency. However, we must admit the significant role of CysC in HF patients with renal dysfunction. In future research, we will choose other perspectives to study on these populations and further discuss the relevant mechanisms.

## 16 Conclusion

Elevated levels of CysC independently and significantly predict adverse outcomes in HF patients with normal to mild renal dysfunction and CHD. Incorporating CysC into the MAGGIC risk score‐based model enhances its prognostic value for predicting adverse outcomes. However, CysC exhibits less prognostic significance for all‐cause mortality in individuals with HFpEF.

## Author Contributions

Study concept and design: Hongbin Liu, Benchuan Hao, and Lyu Lyu. Acquisition of data: Juan Xu, Qiang Yang, Yun Wei, and Lyu Lyu. Analysis and interpretation of data: Benchuan Hao and Lyu Lyu. Drafting of the manuscript: Lyu Lyu, and Juan Xu. Critical revision of the manuscript for important intellectual content: Hongbin Liu, Benchuan Hao, Lyu Lyu, Juan Xu, Qiang Yang, and Yun Wei.

## Ethics Statement

The experimental protocol adhered to the ethical guidelines of the Helsinki Declaration and received approval from the Human Ethics Committee of Chinese PLA General Hospital. Written informed consent was obtained from each participant or their guardian.

## Supporting information


**Supporting File:** clc70310‐sup‐0001‐Additional_file.docx.

## Data Availability

The data sets that were used and evaluated in this study can be the corresponding author upon making a reasonable request.
