# Supplementary material for: Prognostic Value of Cystatin C Across Ejection Fraction Spectrum in Heart Failure With Normal to Mild Renal Dysfunction Original Investigation
Source: Clin Cardiol. 2026 Apr 20;49(4):e70310. doi: 10.1002/clc.70310 (PMC13094363; doi:10.1002/clc.70310)
Supplement: Supplementary file 1 — Supporting File: clc70310‐sup‐0001‐Additional_file.docx. [file CLC-49-e70310-s001.docx]

**Prognostic value of Cystatin C across Ejection Fraction Spectrum in heart failure with normal to moderately impaired renal function**

**Contents:**

1. Table S1. Baseline characteristics of patients according to subtypes of heart failure.

2. Table S2. Uni- and multivariate Cox proportional hazard models for different renal measures as predictors of adverse outcomes.

3. Table S3. Association between different renal measures and adverse outcomes in different subgroups of heart failure.

4. Figure S1. Association between Cystatin C and adverse outcomes using a restricted cubic spline (RCS) regression model.

5. Figure S2. Association between Cystatin C and adverse outcomes in different subgroups of age in HFrEF patients.

**Table S1. Baseline characteristics of patients according to subtypes of heart failure**

| **Baseline characteristics** | **Total**  **(n = 637)** | **HFpEF**  **(n = 235)** | **HFmrEF**  **(n = 236)** | **HFrEF**  **(n = 166)** | ***p*-value** |
| --- | --- | --- | --- | --- | --- |
| Age, median (IQR), years | 66.0 (56.0–75.0) | 74.0 (65.0–82.0) | 59.0 (52.0–70.0) | 62.0 (53.0–71.0) | <0.001 |
| Male, n (%) | 465 (73.0%) | 141 (60.0%) | 191 (80.9%) | 133 (80.1%) | <0.001 |
| Current smokers, n (%) | 183 (28.8%) | 44 (18.7%) | 91 (38.6%) | 48 (29.1%) | <0.001 |
| Body mass index, mean (SD), kg/m² | 24.9±3.5 | 24.6±3.7 | 25.6±3.1 | 24.4±3.6 | 0.001 |
| Systolic blood pressure, median (IQR), mm Hg | 130.0 (117.0–143.0) | 136.0 (124.0–155.0) | 130.0 (117.0–140.0) | 122.0 (110.0–136.0) | <0.001 |
| Diastolic blood pressure, median (IQR), mm Hg | 73.0 (65.0–80.0) | 70.0 (63.0–80.0) | 74.0 (67.0–80.0) | 74.0 (66.0–82.0) | 0.009 |
| Heart rate, median (IQR), bpm | 73.0 (66.0–82.0) | 70.0 (64.0–80.0) | 72.0 (65.0–82.0) | 77.0 (70.0–90.0) | <0.001 |
| NYHA-FC, n (%) |  |  |  |  | 0.001 |
| I/II | 431 (63.4%) | 164 (68.0%) | 177 (68.6%) | 90 (50.9%) |  |
| III | 154 (24.2%) | 57 (24.3%) | 42 (17.8%) | 55 (33.1%) |  |
| IV | 52 (8.2%) | 14 (6.0%) | 17 (7.2%) | 21 (12.7%) |  |
| MAGGIC risk score, median (IQR), point | 19.0 (14.0–25.0) | 22.0 (17.0–27.0) | 16.0 (12.0–20.0) | 19.0 (14.0–25.0) | <0.001 |
| *Medical history* |  |  |  |  |  |
| Diabetes mellitus, n (%) | 241 (37.8%) | 88 (37.4%) | 95 (40.3%) | 58 (34.9%) | 0.550 |
| Hypertension, n (%) | 411 (64.5%) | 190 (80.9%) | 135 (57.2%) | 86 (51.8%) | <0.001 |
| Previous myocardial infarction, n (%) | 209 (32.8%) | 43 (18.3%) | 92 (39.0%) | 74 (44.6%) | <0.001 |
| Previous PCI/CABG, n (%) | 254 (39.9%) | 92 (39.1%) | 95 (40.3%) | 67 (40.4%) | 0.960 |
| Stroke, n (%) | 80 (12.6%) | 40 (17.0%) | 27 (11.4%) | 13 (7.8%) | 0.019 |
| Anemia, n (%) | 15 (2.4%) | 10 (4.3%) | 3 (1.3%) | 2 (1.2%) | 0.054 |
| Chronic obstructive pulmonary disease, n (%) | 58 (9.1%) | 39 (16.6%) | 7 (3.0%) | 12 (7.2%) | <0.001 |
| Atrial fibrillation, n (%) | 81 (12.7%) | 49 (20.9%) | 12 (5.1%) | 20 (12.0%) | <0.001 |
| *Echocardiography* |  |  |  |  |  |
| Left ventricular ejection fraction, median (IQR), % | 46.0 (40.0–55.0) | 57.0 (54.0–60.0) | 45.0 (43.0–47.0) | 36.0 (32.0–38.0) | <0.001 |
| LAD, median (IQR), mm | 39.0 (35.0–42.0) | 38.0 (35.0–41.0) | 37.0 (34.0–41.0) | 41.0 (36.0–45.0) | <0.001 |
| LVPWT, median (IQR), mm | 10.0 (10.0–11.0) | 11.0 (10.0–11.0) | 10.0 (9.0–11.0) | 10.0 (9.0–11.0) | <0.001 |
| LVEDD, median (IQR), mm | 48.0 (45.0–54.0) | 45.0 (42.0–48.0) | 50.0 (46.0–54.0) | 54.0 (49.0–62.0) | <0.001 |
| IVST, median (IQR), mm | 11.0 (10.0–12.0) | 11.0 (10.0–12.0) | 11.0 (10.0–11.0) | 11.0 (9.0–11.0) | <0.001 |
| LVMI, median (IQR), g/m^2^ | 105.4 (90.9–125.7) | 102.3 (88.9–114.7) | 104.7 (89.1–125.0) | 119.8 (98.8–145.0) | <0.001 |
| LAVI, median (IQR), mL/m^2^ | 45.0 (35.1–58.0) | 45.1 (35.7–56.3) | 41.0 (33.2–49.6) | 55.0 (39.6–68.3) | <0.001 |
| LVESD, median (IQR), mm | 36.0 (31.0–41.0) | 31.0 (29.0–34.0) | 37.0 (33.0–41.0) | 43.0 (37.0–50.0) | <0.001 |
| LVEDV, median (IQR), mm | 110.0 (88.0–138.0) | 93.0 (77.0–108.0) | 116.0 (98.0–132.0) | 145.0 (117.0–197.0) | <0.001 |
| LVESV, median (IQR), mm | 58.0 (41.0–81.0) | 39.0 (32.0–48.0) | 63.0 (53.0–76.0) | 94.0 (71.0–130.0) | <0.001 |
| LVFS, median (IQR), mm | 26.0 (21.0–30.0) | 30.0 (28.0–33.0) | 24.0 (22.0–28.0) | 19.0 (16.0–22.0) | <0.001 |
| RVD, median (IQR), mm | 35.0 (32.0–37.0) | 34.0 (31.0–37.0) | 34.0 (32.0–37.0) | 36.0 (32.0–40.0) | <0.001 |
| RAD, median (IQR), mm | 34.0 (31.0–37.0) | 34.0 (30.0–36.0) | 34.0 (32.0–37.0) | 36.0 (32.0–40.0) | <0.001 |
| RVFW, median (IQR), mm | 5.0 (5.0–6.0) | 5.0 (5.0–6.0) | 6.0 (5.0–6.0) | 5.0 (5.0–6.0) | 0.372 |
| IVC, median (IQR), mm | 15.0 (14.0–17.0) | 15.0 (14.0–16.0) | 15.0 (14.0–16.0) | 15.0 (14.0–18.0) | 0.006 |
| E/A, median (IQR) | 0.8 (0.6–1.3) | 0.7 (0.6–1.1) | 0.8 (0.6–1.3) | 0.9 (0.6–1.6) | 0.003 |
| TRV max, median (IQR), m/s | 2.3 (2.1–2.6) | 2.5 (2.2–2.7) | 2.2 (2.0–2.5) | 2.3 (2.1–2.8) | <0.001 |
| AVV max, median (IQR), m/s | 1.2 (1.0–1.4) | 1.3 (1.1–1.5) | 1.1 (1.0–1.3) | 1.1 (1.0–1.2) | <0.001 |
| PAP, median (IQR), mm Hg | 29.0 (22.0–36.0) | 31.0 (28.0–38.0) | 21.0 (19.0–27.0) | 32.0 (27.0–40.0) | <0.001 |
| *Medication* |  |  |  |  |  |
| Statin, n (%) | 592 (92.9%) | 214 (91.1%) | 223 (94.5%) | 155 (93.4%) | 0.337 |
| Beta blocker, n (%) | 496 (77.9%) | 177 (75.3%) | 188 (79.7%) | 131 (78.9%) | 0.489 |
| ACE-I/ARB, n (%) | 330 (51.8%) | 109 (46.4%) | 119 (50.4%) | 102 (61.4%) | 0.010 |
| Diuretic, n (%) | 202 (31.7%) | 59 (25.1%) | 61 (25.8%) | 82 (49.4%) | <0.001 |
| Spironolactone, n (%) | 244 (38.3%) | 55 (23.4%) | 83 (35.2%) | 106 (63.9%) | <0.001 |
| Digoxin, n (%) | 86 (13.5%) | 13 (5.5%) | 21 (8.9%) | 52 (31.3%) | <0.001 |
| Calcium channel blocker, n (%) | 151 (23.7%) | 105 (44.7%) | 30 (12.7%) | 16 (9.6%) | <0.001 |
| *Laboratory indicators* |  |  |  |  |  |
| Creatinine, median (IQR), mg/dL | 0.9 (0.8–1.0) | 0.9 (0.7–1.0) | 0.9 (0.8–1.0) | 0.9 (0.8–1.1) | 0.356 |
| Glucose, median (IQR), mmol/L | 6.4 (5.2–8.5) | 6.2 (5.1–8.2) | 6.8 (5.3–9.2) | 6.4 (5.2–8.4) | 0.031 |
| HbA1c, median (IQR), % | 6.2 (5.7–7.2) | 6.1 (5.7–6.8) | 6.3 (5.7–7.3) | 6.4 (5.8–7.6) | 0.032 |
| HDL-C, median (IQR), mmol/L | 1.0 (0.8–1.2) | 1.1 (0.9–1.3) | 1.0 (0.8–1.2) | 0.9 (0.8–1.1) | <0.001 |
| LDL-C, median (IQR), mmol/L | 2.2 (1.7–2.9) | 2.3 (1.7–2.8) | 2.2 (1.8–2.9) | 2.2 (1.7–2.9) | 0.794 |
| Total cholesterol, median (IQR), mmol/L | 3.8 (3.2–4.5) | 3.8 (3.4–4.4) | 3.8 (3.2–4.7) | 3.8 (3.2–4.5) | 0.440 |
| Triglycerides, median (IQR), mmol/L | 1.3 (0.9–1.8) | 1.2 (0.9–1.6) | 1.3 (0.9–2.1) | 1.2 (0.9–1.6) | 0.015 |
| NT-proBNP, median (IQR), pg/mL | 822.0 (290.0–2,069.0) | 577.0 (265.5–1,253.0) | 709.0 (229.0–1,583.0) | 1,935.0 (691.0–4,310.5) | <0.001 |
| hs-TnT, median (IQR), ng/L | 0.02 (0.01-0.12) | 0.01 (0.01-0.04) | 0.02 (0.01-0.42) | 0.03 (0.01-0.15) | <0.001 |
| eGFR_MDRD_, mean (SD), mL/min/1.73 m^2^ | 99.4±76.2 | 92.8±24.3 | 108.5±120.6 | 95.9±25.5 | 0.065 |
| eGFR_CysC_, mean (IQR), mL/min/1.73 m^2^ | 58.92 (43.79–80.37) | 49.93 (40.09–71.96) | 69.90 (52.82–90.77) | 53.24 (43.70–70.55) | <0.001 |
| cystatin C, median (IQR), mg/L | 1.3 (1.2–1.6) | 1.3 (1.0–1.6) | 1.1 (0.9–1.3) | 1.3 (1.1–1.5) | <0.001 |

HFpEF, Heart failure with preserved ejection fraction; HFmrEF, Heart failure with mid-range ejection fraction; HFrEF, Heart failure with reduced ejection fraction; IQR, Inter-quartile range; NYHA-FC, New York Heart Association functional class; MAGGIC risk score, The Meta-Analysis Global Group in Chronic Heart Failure risk score; PCI, Percutaneous coronary intervention; CABG, Coronary artery bypass grafting; LAD, Left atrium dimension; LVPWT, Left ventricular posterior wall thickness; LVEDD, Left ventricular end-diastolic dimension; IVST, Interventricular septum thickness; LVMI, Left ventricular mass index; LAVI, Left atrial volume index; LVESD, Left ventricular end systolic diameter; LVEDV, Left ventricular end-diastolic volume; LVESV, Left ventricular end-systolic volume; LVFS, Left ventricular fraction shortening; RVD, Right ventricular diameter; RAD, Right atrial diameter; RVFW, Right ventricular free wall; IVC, Inferior vena cava; E/A, early (E) mitral inflow peak/atrial (A) filling peak ratio; TRV, Tricuspid regurgitation velocity; AVV, Aortic valve velocity; PAP, Pulmonary artery pressure; ACE-I, Angiotensin-converting enzyme inhibitor; ARB, Angiotensin II receptor blocker; HbA1c, Hemoglobin A1c; HDL-C, High-density lipoprotein cholesterol; LDL-C, Low-density lipoprotein cholesterol; NT-proBNP, N-terminal pro-brain natriuretic peptide, hs-TnT, high-sensitivity cardiac troponin T; eGFR, estimated glomerular filtration rate; MDRD , Modification of Diet in Renal Disease.

**Table S2. Uni- and multivariate Cox proportional hazard models for different renal measures as predictors of adverse outcomes**

a Model 1 adjusted for age.

b Model 2 adjusted for age; gender; current smoke; BMI; SBP; DBP; heart rate; NYHA-FC; diabetes mellitus; hypertension; previous myocardial infarction; previous PCI/CABG; stroke; anemia; chronic obstructive pulmonary disease; atrial fibrillation; left ventricular ejection fraction; statin; beta blocker; ACE-I/ARB; diuretic; spironolactone; digoxin; calcium channel blocker; glucose; HbA1c; HDL-C; LDL-C; total cholesterol; triglycerides; NT-proBNP, hs-TnT.

HR, Hazard ratio; CI, Confidence interval; eGFR, estimated glomerular filtration rate； MDRD, Modification of Diet in Renal Disease; BMI, Body mass index; SBP, Systolic blood pressure; DBP, Diastolic blood pressure; NYHA-FC, New York Heart Association functional class; PCI, Percutaneous coronary intervention; CABG, Coronary artery bypass grafting; ACE-I, Angiotensin-converting enzyme inhibitor; ARB, Angiotensin II receptor blocker; HbA1c, Hemoglobin A1c; HDL-C, High-density lipoprotein cholesterol; LDL-C, Low-density lipoprotein cholesterol; NT-proBNP, N-terminal pro-brain natriuretic peptide; hs-TnT, high-sensitivity cardiac troponin T.

| **Variables (Log-transformed)** | **Unadjusted HR**  **(95% CI)** | ***p* - value** | **Model 1a**  **Adjusted HR**  **(95% CI)** | ***p* - value** | **Model 2b adjusted HR (95% CI)** | ***p* - value** |
| --- | --- | --- | --- | --- | --- | --- |
| **All-cause mortality** |  |  |  |  |  |  |
| Creatinine | 1.87 (1.01–3.50) | 0.049 | 1.54 (0.82–2.89) | 0.181 | 1.92 (0.89–4.12) | 0.097 |
| Cystatin C | 2.51 (2.13–2.96) | 0.001 | 2.16 (1.73–2.70) | < 0.001 | 5.10 (2.86–9.07) | < 0.001 |
| eGFR_MDRD_ | 0.37 (0.22–0.63) | 0.001 | 0.90 (0.52–1.54) | 0.693 | 0.67 (0.34–1.31) | 0.241 |
| eGFR_CysC_ | 0.50 (0.44–0.56) | 0.001 | 0.56 (0.48–0.66) | < 0.001 | 0.28 (0.18–0.43) | < 0.001 |
| **Heart failure rehospitalization** |  |  |  |  |  |  |
| Creatinine | 3.24 (1.80–5.81) | 0.001 | 1.96 (1.12–3.44) | 0.019 | 2.67 (1.34–5.29) | 0.005 |
| Cystatin C | 2.41 (2.08–2.79) | 0.001 | 1.97 (1.59–2.44) | < 0.001 | 5.04 (3.03–8.38) | < 0.001 |
| eGFR_MDRD_ | 0.21 (0.13–0.34) | 0.001 | 0.55 (0.34–0.90) | 0.016 | 0.39 (0.21–0.72) | 0.002 |
| eGFR_CysC_ | 0.51 (0.46–0.57) | 0.001 | 0.60 (0.51–0.70) | < 0.001 | 0.27 (0.18–0.40) | < 0.001 |

**Table S3. Associations between different renal measures and adverse outcomes in different subgroups of heart failure**

| **Variables (Log-transformed)** | **Unadjusted HR**  **(95% CI)** | ***p* - value** | **Model 1a**  **Adjusted HR**  **(95% CI)** | ***p* - value** | **Model 2b**  **adjusted HR**  **(95% CI)** | ***p* - value** |
| --- | --- | --- | --- | --- | --- | --- |
| **HFpEF** | | | | | | |
| **All-cause mortality** | | | | | | |
| Creatinine | 1.71 (0.63–4.67) | 0.294 | 1.52 (0.51–5.85) | 0.187 | 1.15 (0.54–3.40) | 0.172 |
| Cystatin C | 1.87 (1.44–2.44) | 0.001 | 1.64 (1.13–2.39) | 0.009 | 1.40 (0.92–2.14) | 0.119 |
| eGFR_MDRD_ | 0.46 (0.19–1.07) | 0.070 | 0.81 (0.54–1.18) | 0.263 | 0.59 (0.29–1.20) | 0.144 |
| eGFR_CysC_ | 0.61 (0.51–0.74) | 0.001 | 0.70 (0.52–0.92) | 0.011 | 0.79 (0.57–1.09) | 0.15 |
| **Heart failure rehospitalization** | | | | | | |
| Creatinine | 3.24 (1.80–5.81) | 0.001 | 2.02 (0.73–5.59) | 0.174 | 2.16 (0.78–5.99) | 0.138 |
| Cystatin C | 1.86 (1.50–2.31) | 0.001 | 1.67 (1.26–2.21) | < 0.001 | 1.71 (1.30–2.26) | < 0.001 |
| eGFR_MDRD_ | 0.25 (0.12–0.54) | 0.001 | 0.44 (0.19–0.96) | 0.045 | 0.40 (0.17–0.96) | 0.04 |
| eGFR_CysC_ | 0.62 (0.53–0.73) | 0.001 | 0.68 (0.55–0.84) | < 0.001 | 0.67 (0.54–0.83) | < 0.001 |
| HFmrEF | | | | | | |
| **All-cause mortality** | | | | | | |
| Creatinine | 1.07 (0.34–3.38) | 0.912 | 1.67 (0.52–4.80) | 0.788 | 1.07 (0.15–7.80) | 0.944 |
| Cystatin C | 20.53 (9.92–42.48) | 0.001 | 23.06 (9.55–55.69) | < 0.001 | 10.30 (3.60–29.48) | < 0.001 |
| eGFR_MDRD_ | 0.57 (0.20–1.61) | 0.293 | 0.67 (0.37–1.32) | 0.432 | 0.57 (0.26–1.25) | 0.159 |
| eGFR_CysC_ | 0.10 (0.06–0.17) | 0.001 | 0.09 (0.05–0.18) | < 0.001 | 0.17 (0.07–0.37) | < 0.001 |
| **Heart failure rehospitalization** | | | | | | |
| Creatinine | 1.80 (0.63–5.18) | 0.274 | 1.38 (0.88–2.19) | 0.160 | 1.22 (0.38–3.86) | 0.733 |
| Cystatin C | 13.61 (7.32–25.29) | 0.001 | 7.03 (3.30–14.94) | < 0.001 | 5.61 (2.49–12.61) | < 0.001 |
| eGFR_MDRD_ | 0.29 (0.11–0.77) | 0.012 | 0.81 (0.39–1.66) | 0.555 | 0.78 (0.36–1.66) | 0.516 |
| eGFR_CysC_ | 0.14 (0.09–0.22) | 0.001 | 0.23 (0.13–0.40) | < 0.001 | 0.27 (0.15–0.50) | < 0.001 |
| HFrEF | | | | | | |
| **All-cause mortality** | | | | | | |
| Creatinine | 2.47 (0.88–6.98) | 0.087 | 2.07 (0.81–4.14) | 0.083 | 1.79 (0.84–3.85) | 0.135 |
| Cystatin C | 5.46 (3.19–9.36) | 0.001 | 5.10 (2.39–10.89) | < 0.001 | 2.37 (1.03–2.51) | < 0.001 |
| eGFR_MDRD_ | 0.32 (0.13–0.80) | 0.014 | 0.54 (0.19–1.51) | 0.241 | 0.25 (0.07–0.89) | 0.033 |
| eGFR_CysC_ | 0.23 (0.16–0.35) | 0.001 | 0.27 (0.15–0.46) | < 0.001 | 0.41 (0.18–0.97) | 0.041 |
| **Heart failure rehospitalization** | | | | | | |
| Creatinine | 4.08 (1.52–10.91) | 0.005 | 1.81 (0.62–5.26) | 0.279 | 2.84 (0.76–10.53) | 0.119 |
| Cystatin C | 6.04 (3.57–10.21) | 0.001 | 4.10 (1.73–9.72) | 0.001 | 2.15 (1.09–4.30) | < 0.001 |
| eGFR_MDRD_ | 0.20 (0.09–0.48) | 0.001 | 0.61 (0.24–1.57) | 0.307 | 0.50 (0.17–1.52) | 0.223 |
| eGFR_CysC_ | 0.21 (0.14–0.31) | 0.001 | 0.29 (0.15–0.54) | < 0.001 | 0.45 (0.21–0.97) | 0.043 |

a Model 1 adjusted for age.

b Model 2 adjusted for age; gender; current smoke; BMI; SBP; DBP; heart rate; NYHA-FC; diabetes mellitus; hypertension; previous myocardial infarction; previous PCI/CABG; stroke; anemia; chronic obstructive pulmonary disease; atrial fibrillation; left ventricular ejection fraction; statin; beta blocker; ACE-I/ARB; diuretic; spironolactone; digoxin; calcium channel blocker; glucose; HbA1c; HDL-C; LDL-C; total cholesterol; triglycerides; NT-proBNP, hs-TnT.

HFpEF, Heart failure with preserved ejection fraction; HFmrEF, Heart failure with mid-range ejection fraction; HFrEF, Heart failure with reduced ejection fraction; HR, Hazard ratio; CI, Confidence interval; eGFR, estimated glomerular filtration rate; MDRD, Modification of Diet in Renal Disease; BMI, Body mass index; SBP, Systolic blood pressure; DBP, Diastolic blood pressure; NYHA-FC, New York Heart Association functional class; PCI, Percutaneous coronary intervention; CABG, Coronary artery bypass grafting; ACE-I, Angiotensin-converting enzyme inhibitor; ARB, Angiotensin II receptor blocker; HbA1c, Hemoglobin A1c; HDL-C, High-density lipoprotein cholesterol; LDL-C, Low-density lipoprotein cholesterol; NT-proBNP, N-terminal pro-brain natriuretic peptide; hs-TnT, high-sensitivity cardiac troponin T.

**Figure S1. Association between Cystatin C and adverse outcomes using a restricted cubic spline (RCS) regression model**

**
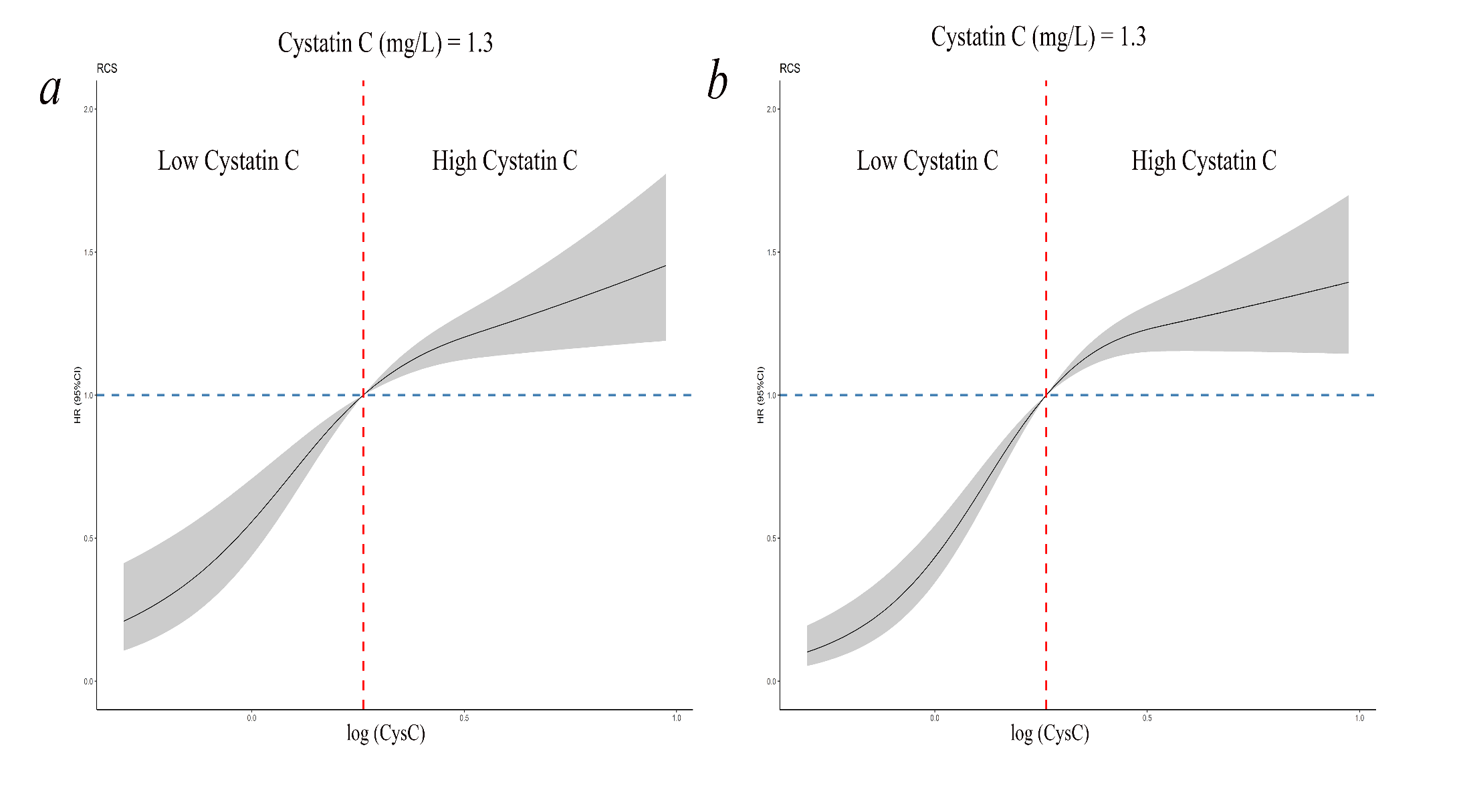
**

Spline curves for a) all-cause mortality and b) HF rehospitalizition.

Spline curves showing the association of Cystatin C as a continuous variable with all-cause mortality (a) and HF rehospitalizition (b). The reference point is the median of Cystatin C.

Spline curves were adjusted for heart rate; MAGGIC risk score; hypertension; previous myocardial infarction; previous PCI/CABG; stroke; anemia; atrial fibrillation; chronic obstructive pulmonary disease; statin; diuretic; spironolactone; digoxin; calcium channel blocker; eGFR_MDRD_; LDL-C; triglycerides; NT-proBNP, hs-TnT.

HF, Heart failure; MAGGIC risk score, The Meta-Analysis Global Group in Chronic Heart Failure risk score; PCI, Percutaneous coronary intervention; CABG, Coronary artery bypass grafting; eGFR, estimated glomerular filtration rate; MDRD, Modification of Diet in Renal Disease; LDL-C, Low-density lipoprotein cholesterol; NT-proBNP, N-terminal pro-brain natriuretic peptide; hs-TnT, high-sensitivity cardiac troponin T.

**Figure S2. Association between Cystatin C and adverse outcomes in different subgroups of age in HFrEF patients**

**
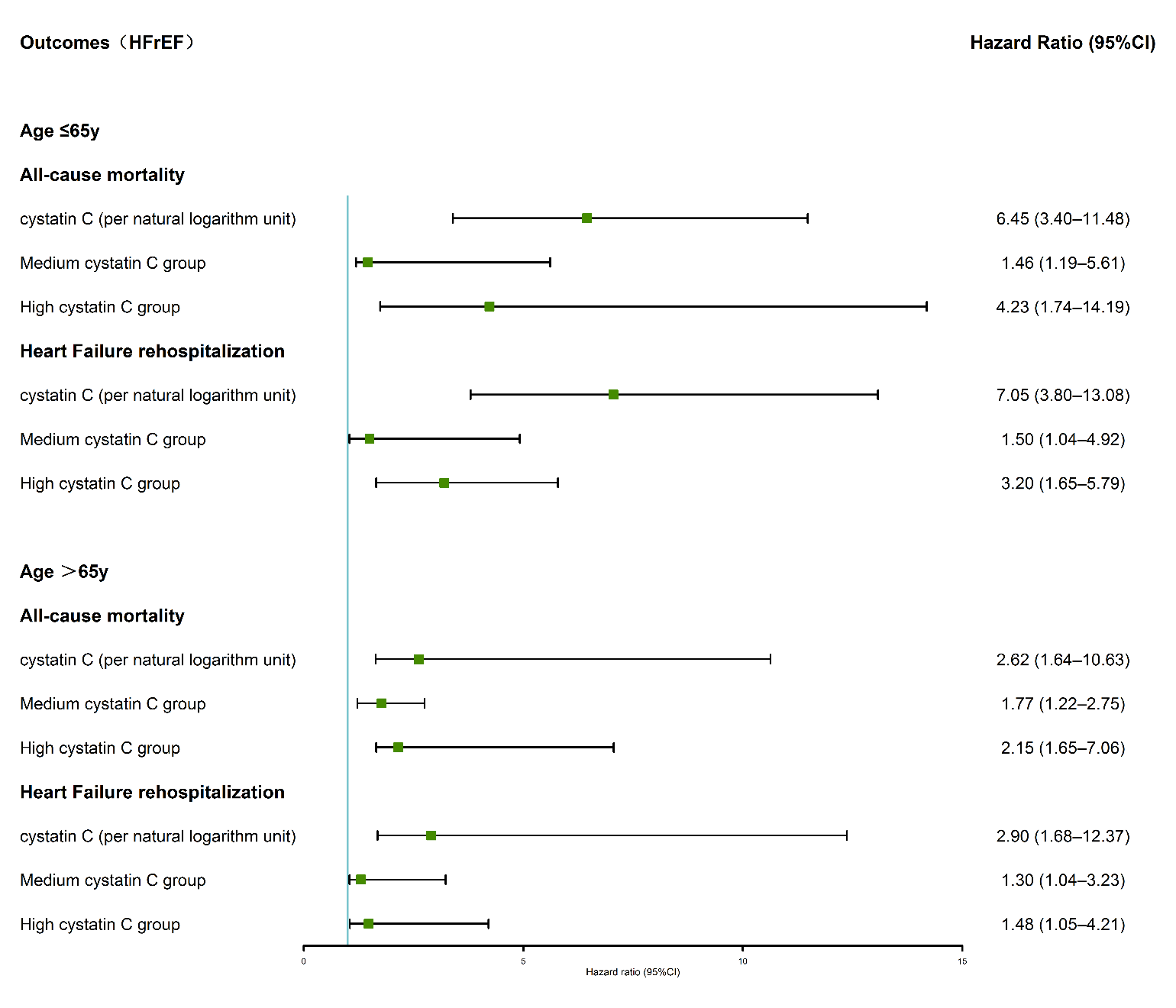
**

Multivariable model adjusted for gender; current smoke; BMI; SBP; DBP; heart rate; NYHA-FC; diabetes mellitus; hypertension; previous myocardial infarction; previous PCI/CABG; stroke; anemia; chronic obstructive pulmonary disease; atrial fibrillation; left ventricular ejection fraction; statin; beta blocker; ACE-I/ARB; diuretic; spironolactone; digoxin; calcium channel blocker; glucose; HbA1c; HDL-C; LDL-C; total cholesterol; triglycerides; eGFR_MDRD_; NT-proBNP, hs-TnT.

HFpEF, Heart failure with preserved ejection fraction; HR, Hazard ratio; CI, Confidence interval; BMI, Body mass index; SBP, Systolic blood pressure; DBP, Diastolic blood pressure; NYHA-FC, New York Heart Association functional class; PCI, Percutaneous coronary intervention; CABG, Coronary artery bypass grafting; ACE-I, Angiotensin-converting enzyme inhibitor; ARB, Angiotensin II receptor blocker; HbA1c, Hemoglobin A1c; HDL-C, High-density lipoprotein cholesterol; LDL-C, Low-density lipoprotein cholesterol; eGFR, estimated glomerular filtration rate; MDRD, Modification of Diet in Renal Disease; NT-proBNP, N-terminal pro-brain natriuretic peptide; hs-TnT, high-sensitivity cardiac troponin T.
